# Effects of Dietary Cholesterol Regulation on Spermatogenesis of *Gobiocypris rarus* Rare Minnow

**DOI:** 10.3390/ijms24087492

**Published:** 2023-04-19

**Authors:** Lv Ye, Mingzhen Zhu, Jian Ju, Hui Yang

**Affiliations:** 1 College of Physical Education, Yangzhou University, Yangzhou 225009, China; 2 College of Animal Science and Technology, Yangzhou University, Yangzhou 225009, China

**Keywords:** cholesterol, spermatogenesis, sex hormone, membrane fluidity, *Gobiocypris rarus*

## Abstract

Cholesterol is an important component of cell membranes, and also a precursor for the synthesis of sex hormones, playing an important role in reproduction. However, few studies have focused on cholesterol and reproductive health. To investigate the toxic effects of different cholesterol levels on the spermatogenesis of rare minnows, we regulate the cholesterol content in fish by feeding them a high-cholesterol diet and cholesterol inhibitor pravastatin, and cholesterol levels, sex hormone (T and 11KT) levels, testis histology, sperm morphology and function, and the expression of genes related to sex hormone synthesis were investigated. The research findings indicate that increasing cholesterol levels significantly increases the liver weight and hepatic–somatic index, as well as the total cholesterol and free cholesterol levels in the testis, liver, and plasma of rare minnow, while inhibiting cholesterol has the opposite effect (*p* < 0.05). However, both increasing and decreasing cholesterol levels can suppress rare minnow testicular development, as evidenced by a decrease in testis weight, lowered gonadosomatic index, suppressed sex hormone levels, and reduced mature sperm count. Further exploration revealed that the expression of sex hormone synthesis-related genes, including star, cyp19a1a, and hsd11b2, was significantly affected (*p* < 0.05), which may be an important reason for the decrease in sex hormone synthesis and consequent inhibition of testicular development. At the same time, the fertilization ability of mature sperm in both treatment groups significantly decreased. Scanning electron microscopy and fluorescence polarization tests showed that reducing cholesterol levels significantly increased the rate of sperm head cell membrane damage, while both increasing and decreasing cholesterol levels led to a reduction in sperm cell membrane fluidity, which may be the main reason for the decrease in sperm fertilization ability. This study demonstrates that both increasing and decreasing the levels of cholesterol are detrimental to the fish spermatogenesis, providing fundamental information for the study of fish reproduction and also a reference for the causes of male reproductive dysfunction.

## 1. Introduction

Cholesterol, an essential lipid, is involved in numerous physiological processes within the body. It plays a critical role in the structure and function of cell membranes, the synthesis of hormones, and the breakdown of fats. Unfortunately, high levels of cholesterol in the bloodstream have been linked to a variety of health issues such as obesity, stroke, and heart disease. Moreover, recent research has shown that disruptions in lipid metabolism can cause reproductive problems. As one of the most cholesterol-rich organs in the body, the testes require cholesterol for proper reproductive function [1].

Previous research indicates that exposure to endocrine disruptors in the environment can cause lipid metabolism disorder, leading to reproductive issues [2]. Lipid metabolism disorder is often closely related to reproductive issues. Cholesterol is especially important in the reproductive process [3]. In the male reproductive system, cholesterol is a precursor to hormone synthesis, regulating the development of the testes and maturation of sperm [4,5]. Additionally, cholesterol is an important component of the cell membrane of germ cells, closely related to membrane fluidity, permeability, and fragility [6]. When cholesterol is deficient, cells become more fragile and prone to rupture [5]. In a survey of normal male populations, there was a highly significant positive correlation between the proportion of normal sperm morphology and sperm cholesterol content [7]. In a survey of male populations with reproductive issues, serum total cholesterol and free cholesterol levels were found to be inversely proportional to the integrity of their sperm acrosome and head size [8]. Therefore, an increasing number of studies suggest that there is a crucial link between cholesterol and male sperm.

Currently, there has been no research conducted on the effects of cholesterol on fish spermatogenesis. To address this gap in knowledge, we utilized *Gobiocypris rarus* rare minnow as our unique model fish for this study. We controlled the fish’s cholesterol levels by adding both cholesterol and the cholesterol inhibitor pravastatin to their feed. We then conducted a comprehensive evaluation of the impact of cholesterol on fish spermatogenesis, including the detection of cholesterol levels, sex hormone levels, testis development, as well as sperm morphology and function. Our research findings will serve as a valuable reference for the protection of fish germplasm resources in aquaculture, as well as provide important insights for investigating male reproductive problems. 

## 2. Results

### 2.1. Changes in Visceral Lipid Deposition and Biological Indicators

**Figure 1:**
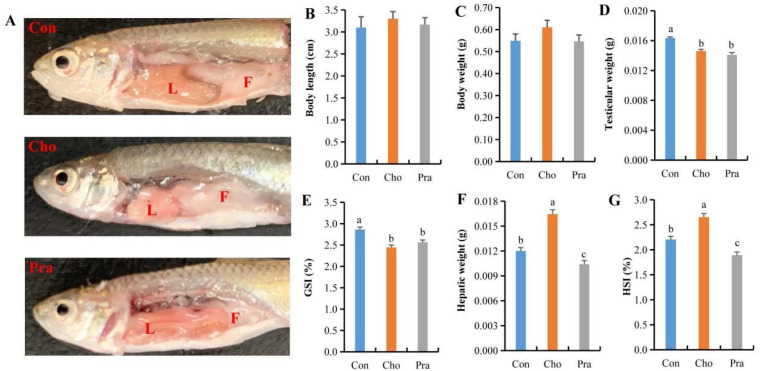
Changes in intraperitoneal lipid deposition and biological indicators of *G. rarus*; (**A**) changes in abdominal fat deposition, L: liver, F: fat; (**B**) body length; (**C**) body weight; (**D**) testicular weight; (**E**) GSI; (**F**) hepatic weight; (**G**) HSI; All statistical data were expressed as mean ± SD. There is a significant difference between the two groups without the same letter at *p* < 0.05 level.

Compared with the control group, *G. rarus* fed with cholesterol had significantly increased visceral fat deposition in the abdomen and showed a whitish liver, while *G. rarus* fed with pravastatin had significantly decreased visceral fat in the abdomen and showed a reddish liver (Figure 1a). The body length and weight of *G. rarus* fed with cholesterol and pravastatin did not significantly differ from those in the control group (Figure 1b,c), but their testis weight and gonadosomatic index (GSI) were significantly lower than those in the control group (Figure 1d,e). The liver weight and hepatosomatic index (HSI) of *G. rarus* fed with cholesterol were significantly higher than those in the control group, while those of *G. rarus* fed with pravastatin were significantly lower (Figure 1f,g).

### 2.2. Changes in Cholesterol Levels and Distribution

**Figure 2:**
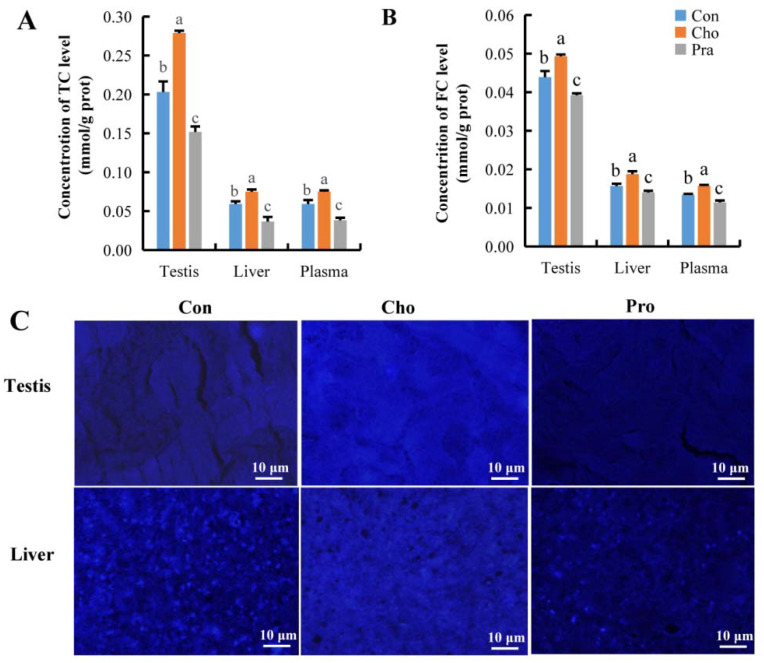
Change in cholesterol level; (**A**) TC; (**B**) FC; (**C**) distribution of FC in testis and liver tissue, blue fluorescence represents FC. All statistical data were expressed as mean ± SD. There is a significant difference between the two groups without the same letter at *p* < 0.05 level.

The measurement results of total cholesterol (TC) and free cholesterol (FC) showed that compared with the control group, *G. rarus* fed with cholesterol had significantly increased TC and FC levels in testis, liver, and plasma, while *G. rarus* fed with pravastatin had significantly decreased TC and FC levels in these organs and plasma (Figure 2a,b). The results of Filipin III staining in testis and liver tissue were consistent with the FC detection results, showing that the FC labeling fluorescence intensity in the testis and liver of *G. rarus* fed with cholesterol was stronger than that in the control group, while the FC labeling fluorescence intensity in *G. rarus* fed with pravastatin was weaker than that in the control group (Figure 2c).

### 2.3. Changes in Testicular Hormone Levels and Developmental Status

**Figure 3:**
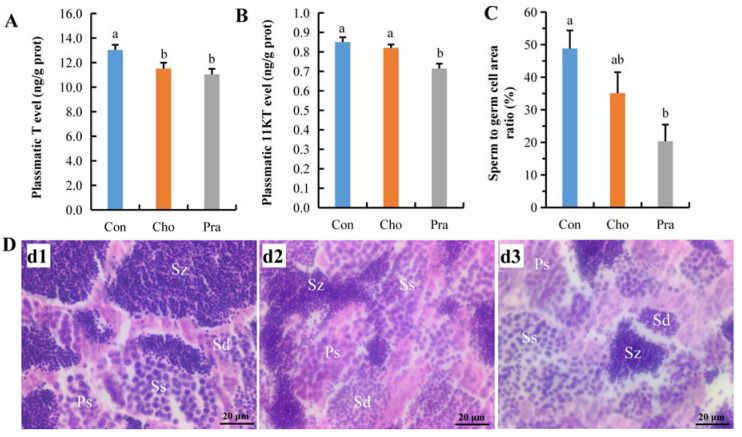
The changes in testis sex hormone levels and the development of testis; (**A**) T; (**B**) 11KT; (**C**) statistical result of sperm to germ cell area ratio; (**D**) paraffin sections of testis; d1: Con, d2: Cho, d3: Pra; Ps: primary spermatocyte; Ss: secondary spermatocyte; Sd: spermatid; Sz: spermatozoa. All statistical data were expressed as mean ± SD. There is a significant difference between the two groups without the same letter at *p* < 0.05 level.

The levels of testosterone (T) in both cholesterol- and pravastatin-fed groups were significantly lower than those in the control group (Figure 3a), and the level of 11 ketol testosterone (11KT) in the pravastatin-fed group was significantly lower than that in the control group (Figure 3b). The paraffin section results showed that the proportion of mature sperm in the pravastatin-fed group was significantly lower than that in the control group, and the proportion of mature sperm in the cholesterol-fed group was also reduced relative to the control group, but it had not yet reached a significant level (Figure 3c,d).

### 2.4. Changes in the Expression of Genes Related to Sex Hormone Synthesis

**Figure 4:**
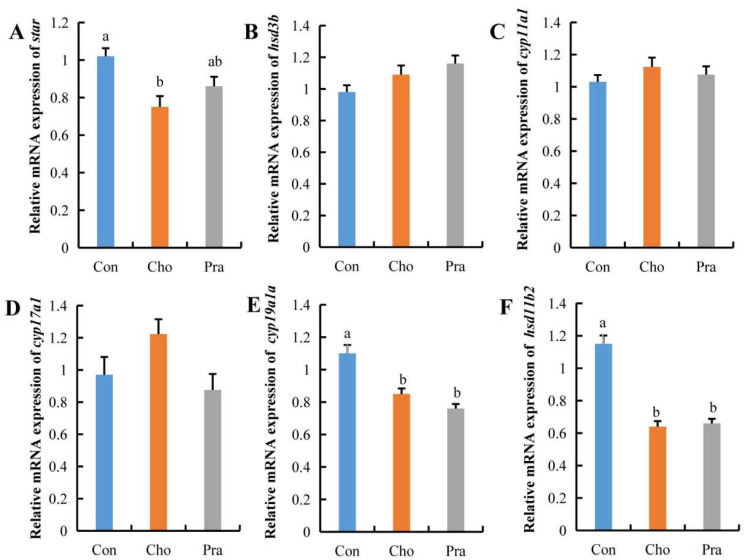
Changes in gene expression related to sex hormone synthesis. All statistical data were expressed as mean ± SD. (**A**–**F**) represents the relative mRNA expression of *star*, *hsd3b*, *cyp11a1*, *cyp17a1*, *cyp19a1a*, and *hsd11b2* genes, respectively. There is a significant difference between the two groups without the same letter at *p* < 0.05 level.

Quantitative real-time PCR (qPCR) results showed that the expression of steroidogenic acute regulatory protein (*star*), which is responsible for transporting cholesterol from outside the cell membrane into the cell, was significantly decreased in the testes of the cholesterol-fed group, while there was a decreasing trend in the pravastatin-fed group. The expression of cytochrome P450, family 19, subfamily A, polypeptide 1a (*cyp19a1a*), and hydroxysteroid (11-beta) dehydrogenase 2 (*hsd11b2*) was significantly decreased in both treatment groups, while the expression of other genes related to sex hormone synthesis showed no significant changes (Figure 4a–f).

### 2.5. Changes in Sperm Cell Membrane and Fertilization Ability

**Figure 5:**
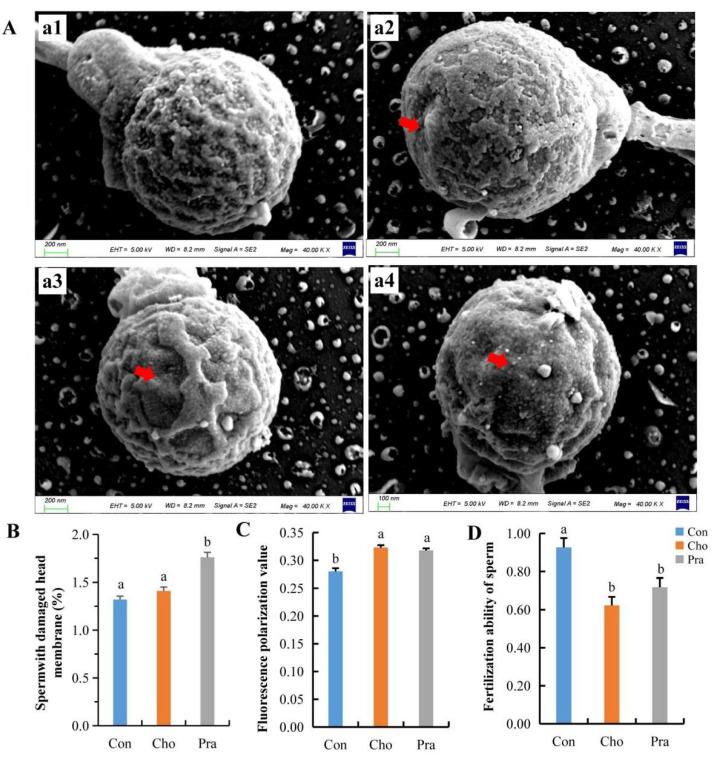
Changes in sperm cell membrane and insemination capacity; (**A**) SEM image of sperm head, a1: normal sperm from Con group, a2: cell membrane defect sperm in Cho group, a3 and a4: cell membrane defect sperm in Pra group, red arrows indicate damaged sperm head membrane; (**B**) cell membrane breakage rate of sperm head; (**C**) fluidity of cell membrane; (**D**) fertilization ability of sperm. All statistical data were expressed as mean ± SD. There is a significant difference between the two groups without the same letter at *p* < 0.05 level.

Scanning electron microscope (SEM) results showed that the sperm head cell membrane in the control group was relatively intact, while the sperm head cell membrane in the cholesterol-fed group showed defects, but the statistical results were not significant. The lovastatin-fed group had significantly more damaged sperm head cell membranes than the control group (Figure 5a,b). Cell membrane fluidity test results showed that the fluorescence values of sperm in both treatment groups were significantly higher than those in the control group, indicating a significant decrease in the fluidity of the sperm head cell membrane in the treatment groups (Figure 5c). The fertilization ability of sperm in both treatment groups significantly decreased (Figure 5d).

## 3. Discussion

The liver is an important site for endogenous cholesterol synthesis and the maintenance of cholesterol homeostasis in animals. Cholesterol homeostasis is one of the important prerequisites for maintaining normal life activities in the body [9]. Fish, as a vertebrate, can rely on endogenous cholesterol synthesis [10]. Cholesterol cannot be completely oxidized and decomposed into water and carbon dioxide in animal bodies, but its side chain can be converted into steroid-like substances through a series of reactions. Testosterone is a male hormone whose synthesis requires the involvement of cholesterol, and excessive cholesterol may also lead to a decrease in testosterone levels [11]. In this study, feeding cholesterol significantly increased the liver weight and HSI of rare minnows, indicating that the experimental treatment was as expected. As a commonly used lipid-lowering drug, pravastatin can reduce total cholesterol, low-density lipoprotein cholesterol (LDL-C), and triglyceride levels in the blood by inhibiting cholesterol synthase, and it can also increase high-density lipoprotein cholesterol (HDL-C) levels and inhibit cholesterol synthesis in the liver [12]. In *G. rarus*, feeding pravastatin significantly reduced liver weight and HSI, which also confirmed the negative regulatory effect of this drug on cholesterol synthesis in fish.

High or low levels of cholesterol may have impacts on testosterone (T and 11KT) levels. Elevated levels of cholesterol may inhibit testosterone synthesis, leading to a decrease in testosterone levels [13]. Conversely, low levels of cholesterol may reduce the supply of cholesterol, thereby affecting testosterone synthesis and physiological function. After treatment with pravastatin, T and 11KT levels also significantly decreased in this study. It was found that the T content in the high-cholesterol group was significantly lower than that in the control group. T is the primary reproductive hormone in male animals, and in fish, T is a precursor to the synthesis of 11KT. Both T and 11KT play important roles in fish spermatogenesis, which does not directly promote the production of sperm but instead promotes sperm maturation and release [14,15,16]. T can also affect the morphology and motility of sperm. Therefore, it is not difficult to understand that the decrease in the proportion of mature sperm is due to a decrease in testosterone content. Previous studies have reported that endocrine disruptors in the environment can also affect fish testosterone levels to varying degrees by disrupting the synthesis, release, transport, and metabolism of endogenous hormones, interfering with the structure and function of androgen receptors, downstream signaling pathways, and transcription factors, thereby changing gene expression and protein synthesis, and affecting reproductive development and behavior in fish. Increasing cholesterol levels may affect sperm production by lowering testosterone. Studies have shown that adding cholesterol to fish feed can increase testosterone synthesis and reproductive ability in fish [17,18,19,20]. Cholesterol may affect fish testosterone synthesis and release by regulating the activity and expression of enzymes such as HMGCR and CYP17 [21,22]. Cholesterol may also affect the structure and function of fish testosterone receptors, thereby affecting their response to and the action of testosterone [23,24]. It should be noted that different species of fish and different physiological states of fish may respond differently to cholesterol, and therefore more in-depth and detailed research is needed to fully understand its mechanisms of action.

Cholesterol is an important component of the cell membrane of reproductive cells, closely related to the membrane’s fluidity and fragility. According to this study, SEM results showed that a decrease in cholesterol levels led to a decrease in the rate of sperm head cell membrane damage, with similar findings observed in a population survey. The lack of cholesterol increases cell fragility and susceptibility to rupture [5]. In a survey of normal male populations, it was found that the proportion of normal sperm morphology was significantly positively correlated with sperm cholesterol content [7]. In a survey of male populations with reproductive problems, it was found that the total cholesterol and free cholesterol levels in the serum were inversely proportional to the integrity of their sperm acrosome [8].

The synthesis of sex hormones in organisms occurs through the use of cholesterol as a precursor. The generation of sex hormones is regulated by the Star, Cyp450, and Hsd [25]. The *star* gene is a transport protein located on the mitochondrial membrane, which transfers cholesterol from the outer membrane to the inner membrane of the mitochondria [26]. This process is the most critical step in synthesizing sex hormones. Currently, the *star* gene has been identified in various aquatic animals, and tissue expression analysis results show that the gene is male-biased [27,28]. Cyp450 is a heme-containing cytochrome superfamily protein, and *cyp19a1a* belongs to this family [29]. It is a crucial enzyme in the steroid synthesis pathway, and it plays a significant role in steroid synthesis, sex determination and differentiation, and gametogenesis [30]. Cyp19 determines the level of sex hormones in the body and has a significant regulatory role in maintaining reproductive system function [31]. With the exception of Hsd17b5 belonging to the aldo-keto reductase (AKR), the remaining 14 subtypes of Hsd17b belong to the short-chain dehydrogenase/reductase (SDR) family [32]. The Hsd17b gene family plays an essential role in the synthesis of steroid hormones and also functions as an oxidoreductase in the final process of sex steroid hormone synthesis [33]. In recent years, it has been proven that Hsd17b is involved in steroid metabolism in aquatic animals such as zebrafish, Japanese eel, *Claris fuscus*, and *Nile tilapia* [34,35]. In *Haliotis diversicolor*, Hsd17b11 plays a significant role in the conversion between testosterone and dihydrotestosterone [36,37]. The *star* gene, *cyp19a* gene, and *hsd11* gene play important roles in male reproductive processes. Both high and low levels of cholesterol significantly inhibit the expression of these genes, thereby adversely affecting the development of male reproductive cells.

In this study, both high and low cholesterol levels were found to affect the motility of sperm cell membranes, thus impacting their fertilization ability. Motility is an important indicator of sperm cell membrane function as it affects the ability and speed of the sperm [38]. Since cholesterol is a major component of sperm cell membranes, it is not difficult to understand how a decrease in its content could lead to membrane damage. Studies have shown that as cholesterol concentrations increase, sperm cell membrane motility decreases, possibly due to cholesterol’s aggregating effect in the membrane [39,40]. However, the effect of cholesterol on sperm cell membrane motility is reversible, with studies showing that a decrease in cholesterol concentration leads to the recovery of membrane motility, possibly due to cholesterol’s dynamic distribution in the membrane.

In conclusion, this study suggests that increasing cholesterol levels significantly increases liver weight, the HSI, and total cholesterol and free cholesterol levels in the testis, liver, and plasma tissues of *G. rarus*, while inhibiting cholesterol has the opposite effect. However, both increasing and decreasing cholesterol levels suppress *G. rarus* testicular development, resulting in a reduced testis weight, decreased GSI, inhibited hormone levels, and decreased mature sperm count. Further exploration reveals that the expression of cholesterol synthesis-related genes *star*, *cyp19a1a*, and *hsd11b2* is significantly affected. Lowering cholesterol levels significantly increases the sperm head cell membrane damage rate, while both high and low cholesterol levels can reduce sperm cell membrane fluidity, which may be the main reason for the decline in sperm fertilization ability. This study proves that both high and low cholesterol levels are detrimental to *G. rarus* sperm production, providing basic information for fish reproduction research, as well as a reference for the causes of male reproductive decline. In the future, further research and exploration are warranted to investigate the effects of different concentrations of cholesterol on male reproductive function.

## 4. Materials and Methods

### 4.1. Feed Preparation and Experimental Fish Rearing

The *G. rarus* used in the experiment were artificially bred in the laboratory and reared in glass tanks with well-aerated water. The water temperature was maintained at 25 ± 0.8 °C and the light/dark cycle was set at 14 h/10 h. The larvae were fed twice a day. After reaching 30 days of age, they were fed twice a day with larvae. After reaching 5 months of age, healthy and uniform-sized male fish were selected for subsequent experiments. Every day, according to the group, the corresponding feed was fed at fixed times and locations twice a day, with a feeding amount calculated as 1.5% of the fish body weight, and three replicates were set. The exposure lasted for 5 weeks, with a water temperature of 25 ± 1 °C and a light/dark cycle of 14 h/10 h. We controlled the cholesterol level in rare cyprinid fish by adding cholesterol (Solarbio, Beijing, China) and cholesterol inhibitor atorvastatin (MedChemExpress, Monmouth Junction, NJ, USA) to the feed. The experiment was divided into three groups: control group (Con), cholesterol group (Cho), and atorvastatin group (Pra). The feed formulation of each group is shown in the table below. Half of the aquaculture water was changed every two days, and residual feed and feces were removed. The feeding test lasted for 30 days. The formulation and composition of experimental diets are shown in Table 1. All experimental procedures were approved by the Animal Ethics Committee of Yangzhou University.

### 4.2. Sample Collection

After the feeding experiment was completed, the experimental fish were anesthetized with MS-222 (500 mg/L, with 200 mg/L NaHCO_3_ as solvent). The surface water was dried with filter paper, and the weight was measured using an electronic balance (accuracy: 0.0001 g), while the total length and body length were measured using a vernier caliper (accuracy: 0.001 cm). Then, the semen was collected using abdominal pressure method. Fresh semen was used for sperm fertilization ability and cell membrane fluidity tests (*n* = 6; 2 fish per replicate), while 2.5% glutaraldehyde was used to fix the semen for scanning electron microscopy sample preparation (*n* = 6; 2 fish per replicate). After semen collection, blood was collected using tail clipping method. Half of the fish’s blood was added to a centrifuge tube containing an anticoagulant, centrifuged to obtain plasma, and then frozen at −80 °C for measurement of T and 11KT levels. The other half of the blood was directly frozen in liquid nitrogen and stored at −80 °C for measurement of total TC and free cholesterol FC levels. After dissection, liver and testis tissues were weighed, and the GSI (GSI = 100 × gonad weight/body weight) and HSI (HSI = 100 × liver weight/body weight) were calculated. Half of the testes from 6 fish (2 per replicate) were fixed in 4% paraformaldehyde. The liver and the other half of the testes from these fish were frozen in liquid nitrogen at −80 °C for frozen section staining of FC. Testes and liver tissues from 6 fish (2 per replicate) were used for measurement of TC and FC levels. The testes from 6 fish (2 per replicate) were used for detection of steroid synthesis-related gene expression. All tissues were frozen in liquid nitrogen and stored at −80 °C. Thirty fish were used in each treatment group, and a total of 90 fish were used in this experiment.

### 4.3. Cholesterol Detection

After grinding the testes, liver, and blood samples with ethanol (approximately 1 mg tissue: 10 μL ethanol), the supernatant was collected by centrifugation for the determination of TC levels (Nanjing Jiancheng, China, A111-1-1; detection limit: 0–10.34 mmol/L) and FC levels (Solarbio, China, BC1895; detection limit: 0.125–6 mmol/L). The precipitate was resuspended in a certain amount of phosphate-buffered saline (PBS) and each protein concentration was determined using a protein quantification kit (Beyotime, Shanghai, China). Finally, the concentrations of TC and FC were normalized to the corresponding sample protein concentrations. FC distribution in testicular and liver tissues was labeled with filipin III, a fluorescent polyene antibiotic that specifically labels FC on cell membranes. First, frozen sections were prepared; after fixing the samples with paraformaldehyde at 4 °C for 24–48 h, they were embedded in OTC and cut into 8–10 μm sections using a cryostat. The sections were mounted on slides, air-dried at room temperature, and then stained. The brief steps for FC staining are as follows: the OTC on the frozen sections was washed away with PBS, and then the sections were incubated with 10 mg/mL Filipin III at room temperature for 2 h. After washing away the excess stain, the sections were mounted with 85% glycerol and observed under a fluorescence microscope.

### 4.4. Measurement of Sex Hormone Levels

Three fish plasma samples were mixed to create each experimental sample. The samples were then diluted with PBS buffer and the plasma T and 11KT levels were measured using the fish T and 11KT assay kit (Shanghai Xinle, Shanghai, China). The protein concentration of each sample was determined using a protein quantification kit, and the sex hormone concentration of each sample was finally normalized to its corresponding protein concentration.

### 4.5. Paraffin Sectioning

After graded ethanol dehydration, testes tissues were washed with xylene and embedded in paraffin. The paraffin-embedded tissues were cut into 6 μm thick sections using a rotary microtome, and the sections were placed in xylene to deparaffinize them. The washed tissues were dehydrated with gradient ethanol. After dehydration, the sections were washed with distilled water and then stained with hematoxylin and eosin (H&E) staining solution. The excess dye was removed by washing with distilled water. The sections were then rehydrated with gradient ethanol and immersed in xylene before being sealed with neutral resin. The sections were observed and photographed under a microscope, and three cross-sections were randomly selected from each section to calculate the ratio of the area of sperm to the area of the cross-section using Image J.

### 4.6. Detection of Gene Expression Related to Sex Hormone Synthesis

Extract total RNA from each sample using Trizol reagent, remove genomic DNA contamination using DNaseI, and detect the degradation degree of total RNA by 1.0% agarose gel electrophoresis. Use Nanodrop1000 micro-nucleic acid detector to measure the ratio of absorbance at 260 nm and 280 nm to detect RNA purity. Precisely quantify sample RNA concentration using the Qubit^®^2.0 fluorescent quantification instrument. The cDNAs were synthesized from total RNA with M-MLV reverse transcription kit (with gDNA wiper) (Vazyme Biotech Co., Ltd., Nanjing, China). Evaluate the mRNA expression of genes related to sex hormone synthesis using qPCR. These genes include *star*, cytochrome P450 family 11 subfamily A member 1 (*cyp11a1*), hydroxy-delta-5-steroid dehydrogenase, 3 beta (*hsd3b*), cytochrome P450, family 17, subfamily A, polypeptide 1 (*cyp17a1*), *cyp19a1a*, and *hsd11b2*. Select actin, beta 1 (*actb*), and eukaryotic translation elongation factor 1 alpha (*ef1a*) as internal reference genes, with primers listed in Table 2. Calculate the qPCR efficiency (E) of each PCR reaction, with E values ranging from 90% to 110%. Use the 2^−∆∆Cq^ method to calculate the relative transcript changes in each gene under BPA exposure.

### 4.7. Observation with Scanning Electron Microscope

After fixation with glutaraldehyde for 48 h, the sample was dropped onto a polylysine-coated slide and left to stand at room temperature until the water was almost evaporated but not completely dry. The sample was then rinsed with PBS and dehydrated with gradient alcohols. Ethyl acetate was used to replace the alcohol and the sample was dried using a critical point dryer. Gold was sprayed (20 nm) and the sample was observed and photographed using a field emission scanning electron microscope. A total of 100 sperm from each fish were observed and the integrity of the sperm head cell membrane was counted.

### 4.8. Detection of Cell Membrane Fluidity

To preserve freshly collected sperm, a sperm preservation solution was used. The cell membrane fluidity of the sperm was tested using the Cell Membrane Fluidity TMA-DPH Fluorescence Polarization Assay Kit (AAT Bioquest, Pleasanton, CA, USA). Proper fluidity of the biological membrane is a necessary condition for normal biological membrane function. Within a certain range, increased membrane fluidity is beneficial for the diffusion and rotational movement of enzyme molecules in the membrane, leading to increased enzyme activity. TMA-DPH is cylindrical and emits fluorescence through dipole transition based on the parallel arrangement of long molecular axes, making it highly sensitive to lipid interactions that cause relocation. According to the instructions of the kit, the brief steps are as follows: suspend the sperm in diluted staining working solution, incubate at 37 °C, wash with PBS, and detect using a fluorescence spectrophotometer with a polarizer set at an excitation wavelength of 355 nm and an emission wavelength of 430 nm. Finally, data are collected for calculation and analysis. The fluidity of the membrane is inversely proportional to the fluorescence value.

### 4.9. Detection of Fertilization Ability

Prior to each fertilization experiment, a batch of 20–30 healthy and uniform-sized female fish was prepared. The eggs from each female fish were mixed using artificial induction, and approximately 100 eggs were placed in each culture dish with six dishes per group. Then, the semen from each male fish was immediately collected and dry fertilization was performed. After fertilization, the fertilized eggs were placed in a constant temperature incubator with light (25.0 ± 0.5 °C) and dead eggs were removed promptly. The fertilization rate was calculated based on the number of surviving eggs at 4 h postfertilization (zygote stage).

### 4.10. Data Analysis

Biological indicators, GSI, and hormone levels were expressed as mean ± standard deviation (mean ± SD). One-way ANOVA was used to analyze the significance of the data. Before the analysis, normality distribution (Shapiro–Wilk) and homogeneity of variance (Levene’s test) were checked. Data that did not meet the normality distribution or homogeneity of variance were logarithmically transformed before performing one-way ANOVA. Tukey post hoc test was used for subsequent analysis of data with significant differences. SPSS Statistics 17.0 was used for analysis.

## Figures and Tables

**Table 1:** Formulation and composition of experimental diets (%).

Feed Ingredients	The Proportion of Each Ingredient (%)		
	Con	Cho	Pra
Fish meal	10	10	10
Soybean meal	18	18	18
Cottonseed meal	18	18	18
Tapioca flour	5	5	5
Soybean oil	4	4	4
Premix ^a^	4.1	4.1	4.1
Lecithin oil	1	1	1
Methionine	0.1	0.1	0.1
Flour	39.8	36.8	39.795
Experimental additive	0	3	0.005
Total	100	100	100

^a^ Premix: vitamin and mineral, respectively (mg/kg): VA: 28 mg, VB1: 12 mg, VB2: 12 mg, VB6: 14.4 mg, VB12: 0.2 mg, VE: 300 mg, VK3: 20 mg, VD: 10 mg, VC: 600 mg, ascorbic acid: 1000 mg, VE: 400 mg, nicotinamide: 80 mg, calcium pantothenate: 100 mg, biotin: 0.4 mg, CuSO_4_·5H_2_O: 10 mg, FeSO_4_·H_2_O: 300 mg, ZnSO_4_·H_2_O: 300 mg, MnSO_4_·H_2_O: 100 mg, Na_2_SeO_3_: 10 mg, CoCl_2_·6H_2_O (10% Co): 2 mg, NaCl: 100 mg, zeolite: 200 mg, MgSO_4_: 500 mg.

**Table 2:** Primers for qPCR.

Gene Name	Primer Name	Primer Sequence 5′ to 3′
*actb*	F	GTCCGTGACATCAAAGAG
	R	ACCGCAAGATTCCATAC
*ef1a*	F	ACAAATGCGGTGGAATCG
	R	TCAAACTTCCAGAGAGCGATA
*star*	F	ACAAATGCGGTGGAATCG
	R	TCAAACTTCCAGAGAGCGATA
*cyp11a1*	F	AGGAGCCCCGAAGGAAAC
	R	ACGACCCATAGCGTACAGACC
*cyp17a1*	F	CTCCCCTCATTGCCTATCAT
	R	TGGGTTTCAGTCAACATCTCAC
*cyp19a1a*	F	CAGTGTGTTTTGGAGATGGT
	R	CTGGACAGATGTGAGTGCTT
*hsd3b*	F	AGTGGTGCTGGCATTGG
	R	TGCTCCTTTACAGGCTCTTC
*hsd11b2*	F	GTTTGGCATCATACGGGGC
	R	TGGGGTTGAGGAGAGAGGAGT

## Data Availability

Data will be available upon reasonable request.
